# Elucidating the Mechanisms of Action of Antimicrobial Agents

**DOI:** 10.1128/mbio.02240-21

**Published:** 2022-04-18

**Authors:** M. Ashley Hudson, Steve W. Lockless

**Affiliations:** a Department of Biology, Texas A&M University, College Station, Texas, USA; The Ohio State University; Ohio State University

**Keywords:** antimicrobial agents, mechanisms of resistance, target deconvolution

## Abstract

Despite the ever-growing antibiotic resistance crisis, the rate at which new antimicrobials are being discovered and approved for human use has rapidly declined over the past 75 years. A barrier for advancing newly identified antibiotics beyond discovery is elucidating their mechanism(s) of action. Traditional approaches, such as affinity purification and isolation of resistant mutants, have proven effective but are not always viable options for identifying targets. There has been a recent explosion in research that relies on profiling methods, such as thermal proteome profiling in bacteria, for better understanding the mechanisms of discovered antimicrobials. Here, we provide an overview of the importance of target deconvolution in antimicrobial discovery, detailing traditional approaches, as well as the most recent advances in methodologies for identifying antimicrobial targets.

## THE ANTIBIOTIC RESISTANCE CRISIS

Since the discovery of antibiotics, the number of deaths attributed to infectious disease has decreased by 70% (1). However, microorganisms have evolved powerful mechanisms to cope with antibiotic exposure, and development of resistance to a single antibiotic is nearly inevitable. In addition to acquired resistance, many microorganisms are inherently resistant to many of the antibiotics available for treating infections. As a result, antibiotic-resistant pathogens are responsible for at least 700,000 deaths each year globally (1). Driven by factors such as misuse and overuse, the crisis is only worsening, and the World Health Organization has classified antimicrobial resistance as one of the top 10 global health threats faced by humanity (2). Despite decades of worsening figures, there has been a well-documented decrease in new antibiotics in the discovery pipeline, particularly of first-in-class antibiotics that target novel bacterial processes (3, 4). In fact, since the identification of penicillin in 1928, there have been only a few physiological processes targeted by antibiotics, and newly approved antibiotics are often derivatized versions of these major classes.

To address the urgent need for new antibiotics, researchers have turned to screening libraries of diverse natural and synthetic molecules, where two major screening methodologies prevail (5, 6). In biochemical screening, molecules are tested for activity against a validated bacterial target, such as an essential protein, so the target is well established prior to the screen being conducted. However, there has been a recent shift to phenotypic-based screening, where a parameter of cellular function is measured in response to screening molecules. An example includes identifying antimicrobial molecules that alter ATP synthesis, where the observed phenotype during screening is the concentration of intracellular ATP in bacterial cells (7). Disruption of numerous cellular pathways could lead to reduced ATP, so experiments to reveal the precise target(s) occur after lead molecules have been identified. Phenotypic-based screens have uncovered promising antimicrobial agents, but a key hurdle for advancing these molecules in the development pipeline is this task of elucidating the molecular mechanisms of action (MOA) responsible for their activity. Also important for validating their use as therapeutics is determining whether antimicrobial agents target processes unrelated to their antimicrobial activity. Therefore, it is critical to have sound methodologies for understanding both the targets (the precise binding partner of a molecule) and MOAs (the broad physiological process they disrupt) of new antimicrobial agents.

## IMPORTANCE OF MECHANISTIC INSIGHTS

Although not a requisite for FDA approval, understanding an antimicrobial’s MOA is useful for its development beyond discovery (8–10). Clinical trials are both expensive and cumbersome and are more likely to fail if mechanistic insight is lacking. For example, a mechanistic insight can help predict the spectrum of activity across microorganisms. This information can also be used to strategically derivatize molecules to lessen host toxicity, increase affinity, or promote uptake. Methodologies for characterizing MOAs include biochemical approaches, such as affinity chromatography or thermal proteome profiling, which can both be used to detect direct protein-ligand interactions (11–13). Genetic approaches, such as selecting and screening for resistance, are also widely used, and with the advent of RNA sequencing, transcriptome changes can be monitored to identify global expression patterns (14–16). Other methodologies rely on generating antibiotic signatures for known antibiotics and comparing them to those with unknown MOAs, for example, by generating morphological or metabolic profiles (17–19). Each of these approaches, including discussions of their advantages and shortcomings, is described below, and examples of their uses are cited where applicable (Table 1).

**TABLE 1:** Summarized methodologies for elucidating MOAs

Approach	Advantage(s)	Disadvantage(s)	References
Affinity chromatography	Identifies direct biophysical interactions	Requires ligand immobilization, detects only high-affinity interactions, requires abundant targets	24, 51, 52
Macromolecular synthesis	Reliably identifies alterations in macromolecular biosynthesis pathways	Only classifies MOAs as disrupting known macromolecular biosynthesis pathways	22, 53
Resistance selection	Does not require specialized equipment, can identify precise target(s)	Resistance does not always easily arise, resistance not always due to mutations in target	27, 28, 54
Resistance screening	Does not require specialized equipment, can identify precise target(s), has high-throughput capabilities	Limitations on genetic tools available for some microorganisms	39, 44
Thermal proteome profiling	Can identify precise target(s), does not require ligand immobilization	Detects only high-affinity interactions, high cost	13, 46
Signature methodologies	Reliably classifies into broad MOA, has high-throughput capabilities	Only identifies previously described MOAs, can be time-consuming and costly to generate a wide range of signatures for known antibiotics	17, 18, 47, 55

## TRADITIONAL BIOCHEMICAL AND GENETIC APPROACHES

### Effects on macromolecular synthesis.

Most antibiotics can be classified into one of a few categories based on the key biosynthetic processes they disrupt, such as those that target DNA, RNA, proteins, or peptidoglycan synthesis. The effect of antimicrobials on these macromolecular synthesis pathways can be measured to infer MOAs. This is achieved through direct quantification of their biosynthesis, typically by measuring the proportion of radiolabeled precursors incorporated over time into each of these structures in antimicrobial-treated cells (20, 21). For example, the MOA of teixobactin, a natural product that targets peptidoglycan synthesis in multidrug-resistant Gram-positive bacteria, was discovered using this approach (22).

Approaches that classify an antimicrobial into a known MOA category have the obvious drawback that they will not identify novel MOAs, i.e., ones that target new proteins or pathways. It is also difficult to establish whether the changes in biosynthesis rates of known pathways are due to direct action of the antimicrobial or indirect effects due to growth inhibition. Nevertheless, a time course experiment can often provide valuable information that narrows down the MOA, which can be followed up with more detailed studies.

### Affinity chromatography.

One of the most widely used purification methods, affinity chromatography is a longstanding approach for identifying interactions between antimicrobials and their targets. Notable uses include the discovery of penicillin’s interaction with penicillin-binding proteins and vancomycin’s binding to the d-Ala-d-Ala terminus of peptidoglycan precursor lipid II (23–25). In affinity chromatography, ligands of interest (i.e., the antimicrobial agent) are immobilized to a physical matrix, and proteins are isolated from whole-cell extracts by binding to the immobilized ligand. Subsequent washes remove nonbound components, and the molecule of interest is eluted. Purified binding molecules are then identified using analytical methods such as mass spectrometry. In addition to identifying targets responsible for a given molecule’s activity, if there is sufficient binding, affinity purification can also be used to identify undesired targets of antimicrobials.

Affinity chromatography is not without its limitations. For small molecules and other antimicrobial agents, immobilization is typically achieved through creating a covalent bond from a ligand functional group to a reactive matrix (11, 26). This often obstructs the antimicrobial agent’s activity, rendering it ineffective for target pulldown. Affinity chromatography also requires an abundance of target in the extract, which means it is not practical for agents that bind to low-abundance proteins. Additionally, affinity purification requires fairly strong, long-lasting interactions between a ligand and its target(s). Due to the often stringent wash steps, larger protein complexes can be disrupted, which makes it more difficult to identify targets that are multiprotein complexes. Additionally, many antibiotics do not target specific proteins but instead interact with other components of the cell; for example, daptomycin targets the cytoplasmic membrane of Gram-positive bacteria, and colistin targets the lipopolysaccharide of Gram-negative bacteria. Such targets would not be easily identified using affinity purification methods. Despite its limitations, affinity purification is an attractive target deconvolution approach because it relies on physical interactions to detect the direct molecular targets, which can be used to rationally design antimicrobial derivatives.

### Selecting for resistance.

Bacteria are continuously evolving, so exposure to antibiotics will ultimately select for resistant mutant strains. While bacteria deploy an arsenal of resistance mechanisms, a common one is genetic modification to the target itself. This can be exploited to identify an antimicrobial’s molecular target by exposing bacteria to an antimicrobial, selecting for resistance, and identifying causal mutations. Typically, mutations will not only arise in the gene encoding the target but in the binding site itself, providing valuable information about important amino acids involved in the interaction. It is also a valuable way to identify off-target binding (i.e., molecule binding to proteins unrelated to the principal antimicrobial activity of the molecule.) With the growing accessibility of bacterial whole-genome sequencing, this is one of the most reliable approaches for determining an antimicrobial agent’s mechanism.

A well-characterized example of this is the identification of the target of rifampin, a transcription-disrupting antibiotic. First shown in Mycobacterium tuberculosis, rifampin resistance is attributed to single nucleotide polymorphisms in *rpoB*, the gene that encodes the β-subunit of RNA polymerase (27). Also determined using resistance selection was the target of bedaquiline, a diarylquinoline antibiotic that kills Mycobacterium tuberculosis. Bedaquiline-resistant strains exhibit readily identifiable mutations in the gene encoding AtpE, a component of the F_0_ subunit of ATP synthase, leading researchers to uncover this as the target (28).

While selecting for resistance is generally straightforward, the variety of resistance mechanisms means that resistance-conferring mutations can be unrelated to the direct target. For example, a bacterium with an increased efflux of the molecule would become resistant, but the mutations arising would not be in the direct target (29). In such a case, one can select for resistant colonies at different compound concentrations, with the most resistant typically having mutations in the target. Additionally, resistance does not always easily arise, which may be an enticing feature for antimicrobial development but complicates target identification using selection-based methodologies. Fortunately, there are approaches for increasing resistance rates, thereby increasing the chance that a favorable mutation (or sets of mutations) will arise in the genome. Ethyl methane sulfonate is commonly used to induce guanine alkylation, increasing resistance frequency to antibiotics such as rifampin by approximately 4 orders of magnitude (30). Furthermore, serially passaging cultures at a fixed concentration can select for slow-growing resistant cells, and exposure to increasing concentrations over time can lead to the evolution of resistant strains (31). It should be noted that antibiotic concentration in such experiments can impact the mechanism of resistance (32). Methods may still fail to give rise to selectable resistance, especially in cases where the targets are highly conserved structures. In cases where resistance does not readily arise, screening for resistance in large genetic libraries may be more fruitful.

### Resistance screening.

There are many large-scale mutant libraries available for commonly used bacterial species, which, in combination with high-throughput capabilities, allow for implementation of large-scale resistance screens. These tools can be used to elucidate antimicrobial targets in the absence of easily selectable mutants, namely, by probing the susceptibility of library strains to molecules of interest. Deletion libraries, such as the Keio collection in Escherichia coli or the Nebraska transposon mutant library in Staphylococcus aureus, can be screened to identify disruptions that lead to an altered response to an antimicrobial agent (33, 34). For example, Nichols et al. screened an E. coli single-gene deletion library for phenotypic changes in response to numerous well-known antibiotics, allowing for novel insights into MOAs (35). Similarly, transposon sequencing can be used for this same purpose (36). For example, Geisinger et al. utilized transposon sequencing to screen Acinetobacter baumannii mutants for antibiotic susceptibility across a wide range of antibiotics (37).

Deletion libraries, however, do not contain strains with disruptions of essential genes unless the gene is conditionally essential, i.e., the environmental conditions influence the essentiality of a gene, or unless additional mutations are present that suppress the deletion phenotype. Therefore, if a molecule targets an essential protein, as most antimicrobials do, it would not be uncovered using a collection of gene deletion strains. To circumvent this, overexpression libraries, such as the E. coli ASKA library, can be used to identify high-copy suppressors of growth inhibition (38). For example, Pathania et al. utilized a high-expression clone library for essential genes in E. coli and found that overexpression of LolA, a protein involved in lipoprotein trafficking, led to resistance to a novel antimicrobial agent, MAC13243 (39). Likewise, with the introduction of CRISPR technology, CRISPR interference libraries have been successful in identifying essential targets. In contrast to overexpression libraries, libraries in which essential genes are depleted via decreased expression or function (termed hypomorphs) can be screened for increased sensitivity to the antibiotics (40). There are several examples in which this methodology has proven successful, including work identifying targets in Mycobacterium tuberculosis and Staphylococcus aureus (41–43). A drawback to screening methodologies is that while these libraries exist for numerous bacterial species, many still lack genetic tools to carry out such experiments (44). Despite this, isolating resistant mutants through either mutant screening or selection offers a straightforward approach for identifying antimicrobial targets, and it remains one of the most widely used methods to date.

## RECENT ADVANCES: A SHIFT TO PROFILING METHODOLOGIES

### Thermal proteome profiling.

First described in human cells, thermal proteome profiling (TPP) has revolutionized drug target identification. Its basis relies on a classic thermodynamics principle—the thermal stability of a protein increases when bound to ligand. Several thermal shift methodologies, including the cellular thermal shift assay (CETSA), utilize this principle to characterize protein interactions (12, 45). However, when combined with multiplexed quantitative mass spectrometry using tandem mass tags, TPP’s advantage is that it enables a proteome-wide view of thermal stability.

**FIG 1:**
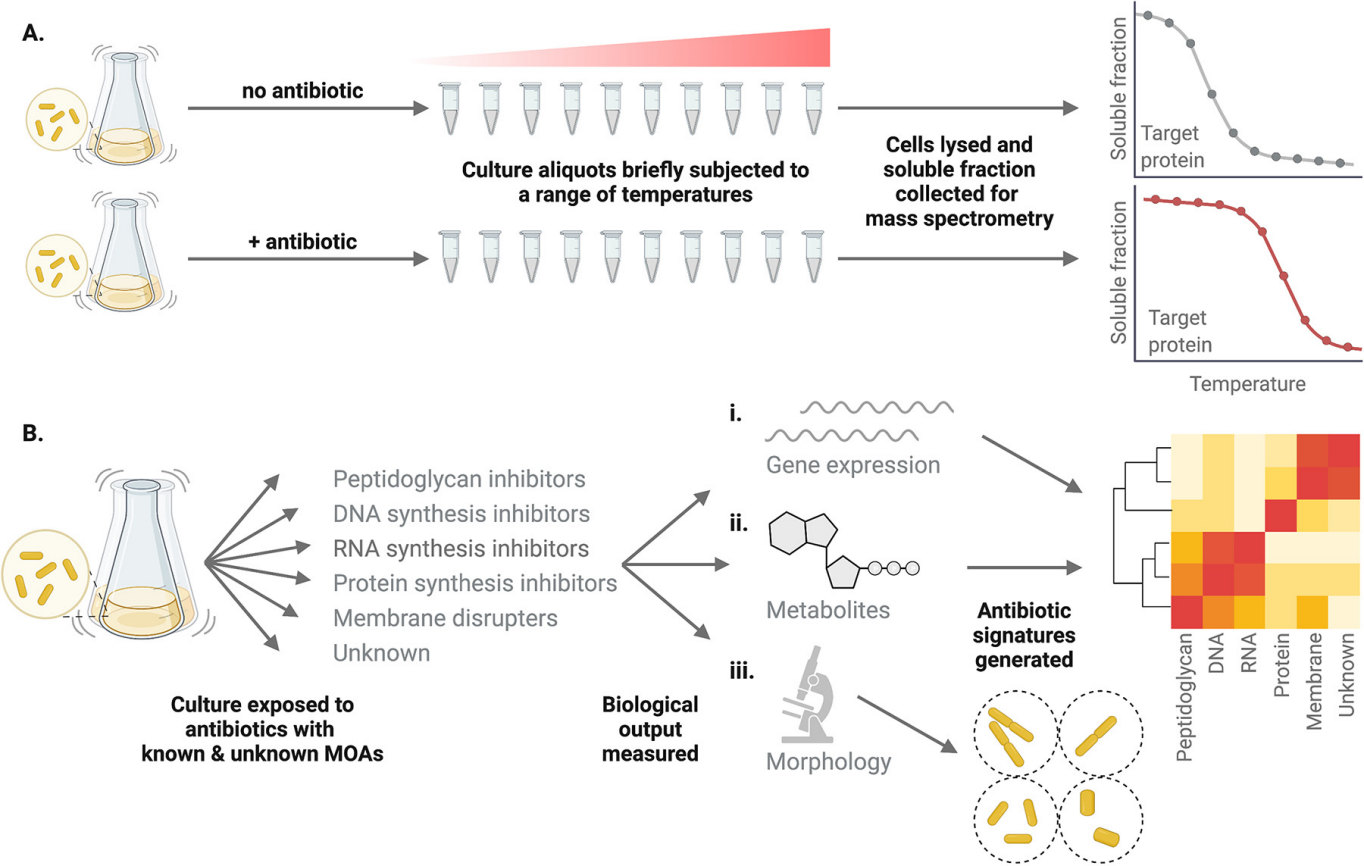
Novel approaches rely on profiling methodologies. (A) In thermal proteome profiling, cultures are heated to a range of temperatures in the presence and absence of the ligand of interest. Cells are then lysed, and the soluble fraction is collected and subjected to multiplexed quantitative mass spectrometry using tandem mass tags. Profiles for each protein characterized are then be compared in the presence and absence of the antimicrobial agent to identify those with altered thermal stability. Proteins with increased thermal stability in the presence of the antimicrobial are likely the target protein. (B) In methodologies relying on antibiotic signatures, bacterial cultures are exposed to antibiotics with known MOAs and a phenotypic response, such as (i) gene expression, (ii) metabolite concentration, or (iii) cellular morphology, is recorded. The responses of cultures treated with antibiotics with unknown MOAs are then compared to those with known MOAs. For transcriptomic and metabolomic data, this typically includes generating clusters of differentially expressed genes or metabolites to infer MOA. For cytological profiling, bacterial morphological profiles are established.

TPP is performed by heating aliquots of a cell culture to a range of temperatures in the presence and absence of the ligand of interest (i.e., the antimicrobial agent). The original protocol for performing this technique in E. coli utilizes 10 different temperatures, and for each temperature, the proteome is characterized quantitatively using mass spectrometry (45). An antimicrobial binding to a target would increase the melting temperature, leading to increased stability, while proteins not bound to ligand would denature and sediment at the same temperature. Therefore, proteins that exhibit increased stability in the presence of the antimicrobial agent are target candidates (Fig. 1A).

In 2018, the first proof-of-principle study published for bacteria was used to confirm the targets of two antibiotics with previously characterized targets. In ampicillin-treated cultures, TPP successfully identified a few proteins with increased stability, including the known penicillin binding protein targets; it also identified resistance-mediating β-lactamases, highlighting that it can successfully identify binding partners other than the direct target (45). Likewise, when treated with ciprofloxacin, a DNA gyrase-targeting antibiotic, the GyrB subunit of DNA gyrase was thermally stabilized. Though new, this technology has already been used to successfully detect targets for novel antimicrobial agents. SCH-79797, a broad-spectrum antimicrobial identified through a high-throughput growth inhibition screen, was found to shift the thermal stability of dihydrofolate reductase, and subsequent experiments confirmed this was indeed its target (13). TTP was also used to detect the target of the antiparasitic agent ENH1, where it identified thermal shifts of calcium-dependent protein kinase 1 (CDPK1) in *Toxoplasma* (46).

Like affinity chromatography, TPP has the advantage of detecting direct biophysical interactions to identify direct targets, that is, the binding partner of the agent that ultimately leads to cell death, as well off-targets, any interactions that take place but are unrelated to the antimicrobial action. Unlike affinity chromatography, however, TPP does not require immobilization of the agent, and the binding of target to ligand occurs in intact cells. While evidence of its utility in target identification is apparent, TPP requires specialized equipment and technical expertise, leading to high cost. It may also have difficulty identifying low-affinity interactions. For example, with ciprofloxacin, an insignificant thermal shift of the GyrA subunit of DNA gyrase occurred despite there being a well-characterized interaction between the two (45). Additionally, like affinity purification, TPP can only recognize protein targets, and not all antibiotics target proteins. Nevertheless, though still in its infancy, it is gaining traction as a steadfast method for accurately identifying targets of antimicrobial agents.

### Antibiotic signatures.

In methodologies relying on antibiotic signatures, phenotypic parameters are established for bacteria treated with antibiotics, and the antimicrobial in question is compared to those with established mechanisms (Fig. 1B). Rather than reporting on the precise target, it identifies am MOA category based on profiles of antibiotics with known MOAs. Described below are profiling examples based on transcriptomes, metabolomes, and cellular morphology.

### (i) Antibiotic signatures: bacterial cytological profiling.

Bacteria treated with different antimicrobials display a wide range of morphological changes dependent on their MOA. By staining distinct cellular features in antibiotic-exposed bacteria, morphological profiles can be established for antibiotics with known MOAs and then compared to those generated by exposure to antibiotics with unknown mechanisms. For example, in the work pioneering bacterial cytological profiling (BCP), cellular membranes and DNA were stained with FM4-64 and DAPI (4′,6-diamidino-2-phenylindole), respectively, and morphological characterization of these two structures after antibiotic exposure established five major categories of inhibitors, including those that disrupt biosynthesis of DNA, RNA, protein, peptidoglycan, and lipids (17). Within these five categories, the authors were also able to distinguish subcategories, including, for example, three subcategories of protein synthesis inhibitors.

In its founding study, BCP was used to determine the MOA for spirohexenolide A, a natural product with broad-spectrum activity against bacteria, as well as human cells. Spirohexenolide A’s cytology profile closely resembled that of nisin, an antibiotic that binds lipid II, forming membrane pores and inhibiting cell wall synthesis, thereby collapsing the proton motive force in susceptible bacteria (17). Follow-up assays demonstrated that both spirohexenolide A and nisin led to permeabilization of the E. coli membrane and collapsed proton motive forces, suggesting cytological profiling accurately classified spirohexenolide A’s mechanism. It was also used to identify the MOA for nonsteroidal anti-inflammatory (NSAIDs) antibiotics, such as carprofen and flufenamic acid (18). Contrary to a recent study characterizing the bacterial target of NSAID antibiotics as DnaA, BCP found morphological changes in B. subtilis consistent with membrane disrupters, highlighting its utility in identifying off-target effects on bacterial physiology.

BCP can easily be performed in high throughput, where generating antibiotic profiles can be quite efficient. As with other methodologies that rely on antibiotic signatures, a drawback is its inability to identify MOAs not previously characterized. There have been attempts to circumvent this, including the advent of rapid inhibition profiling (RIP), which generates morphological profiles based on degradation of essential proteins, rather than exposure to antibiotics, has proven effective (18). Using RIP, profiles of new antibiotics can be compared to the profiles of those with degraded essential proteins to identify targets and pathways involved in inhibition.

### (ii) Antibiotic signatures: global transcriptome profiling.

Using DNA microarrays and RNA sequencing, gene expression studies have enabled glimpses into the response of bacteria to antibiotic (47–49). Like with morphology in bacterial cytological profiling, transcriptome signatures can be generated across a wide range of antibiotics to experimentally classify an unknown antimicrobial’s MOA. Many of these studies have established that transcriptomic changes are often not directly related to an antimicrobial’s target because exposure to a given antimicrobial leads to global effects that are dependent on both duration of exposure and molecule concentration. Since gene expression changes are often representative of downstream adaptations, bacteria treated with known antibiotics tend to have global expression profiles that cluster together. Additionally, transcriptome profiling is not effective at identifying mechanisms for first-in-class antibiotics, and unless the organism of interest already has antibiotic profiles generated for it, profiles will need to be experimentally generated.

### (iii) Antibiotic signatures: metabolomic fingerprinting.

Like cytological and transcriptomic profiling, metabolic fingerprinting relies on generating metabolomic profiles of antibiotic-treated bacteria using various spectroscopic method methods, such as mass spectroscopy, nuclear magnetic resonance spectroscopy, or Fourier transform infrared spectroscopy (19, 50). This approach offers a powerful and high-throughput tool for rapidly classifying MOAs. For example, metabolomic fingerprinting was used to build profiles of Mycobacterium smegmatis upon treatment with 62 antimicrobial agents with known MOAs. Using these profiles, over 100 antimycobacterial molecules with unknown mechanisms were able to successfully be classified among these categories, highlighting its ability to be used to generate an abundance of information for molecules in which we are deficient in mechanistic data (50).

## CONCLUSIONS

There is a profound lack of new antibiotics being employed to combat multidrug-resistant bacteria. Several recent studies have detailed the discovery of promising antimicrobial compounds, but these molecules rarely advance to clinical use. For many of these molecules, we do not have a clear understanding of their targets, which significantly hinders them from proceeding in the development pipeline and gaining regulatory approval. Recent publications have described novel approaches to confront the problem of characterizing a molecule’s MOA, especially for those in which traditional approaches fail. There has been a general shift to using profiling methodologies. Here, we provided an outline of these novel approaches, along with a brief overview of established methodologies.

## References

[1] Plackett B. 2020. No money for new drugs. Nat Outlook 586:S50–S52. doi:10.1038/d41586-020-02884-3.

[2] World Health Organization. 2022. Antimicrobial resistance. https://www.who.int/news-room/fact-sheets/detail/antimicrobial-resistance.

[3] Luepke KH, Suda KJ, Boucher H, Russo RL, Bonney MW, Hunt TD, Mohr JF. 2017. Past, present, and future of antibacterial economics: increasing bacterial resistance, limited antibiotic pipeline, and societal implications. Pharmacotherapy 37:71–84. doi:10.1002/phar.1868. 27859453

[4] Jackson N, Czaplewski L, Piddock LJV. 2018. Discovery and development of new antibacterial drugs: learning from experience? J Antimicrob Chemother 73:1452–1459. doi:10.1093/jac/dky019. 29438542

[5] Miethke M, Pieroni M, Weber T, Brönstrup M, Hammann P, Halby L, Arimondo PB, Glaser P, Aigle B, Bode HB, Moreira R, Li Y, Luzhetskyy A, Medema MH, Pernodet JL, Stadler M, Tormo JR, Genilloud O, Truman AW, Weissman KJ, Takano E, Sabatini S, Stegmann E, Brötz-Oesterhelt H, Wohlleben W, Seemann M, Empting M, Hirsch AKH, Loretz B, Lehr CM, Titz A, Herrmann J, Jaeger T, Alt S, Hesterkamp T, Winterhalter M, Schiefer A, Pfarr K, Hoerauf A, Graz H, Graz M, Lindvall M, Ramurthy S, Karlén A, van Dongen M, Petkovic H, Keller A, Peyrane F, Donadio S, Fraisse L, et al. 2021. Towards the sustainable discovery and development of new antibiotics. Nat Rev Chem 5:726–749. doi:10.1038/s41570-021-00313-1. 34426795 PMC8374425

[6] Tommasi R, Brown DG, Walkup GK, Manchester JI, Miller AA. 2015. ESKAPEing the labyrinth of antibacterial discovery. Nat Rev Drug Discov 14:529–542. doi:10.1038/nrd4572. 26139286

[7] Mak PA, Rao SPS, Ping Tan M, Lin X, Chyba J, Tay J, Ng SH, Tan BH, Cherian J, Duraiswamy J, Bifani P, Lim V, Lee BH, Ling Ma N, Beer D, Thayalan P, Kuhen K, Chatterjee A, Supek F, Glynne R, Zheng J, Boshoff HI, Barry CE, Dick T, Pethe K, Camacho LR. 2012. A high-throughput screen to identify inhibitors of ATP homeostasis in non-replicating Mycobacterium tuberculosis. ACS Chem Biol 7:1190–1197. doi:10.1021/cb2004884. 22500615 PMC3401038

[8] Anonymous. 2010. Mechanism matters. Nat Med 16:347. doi:10.1038/nm0410-347. 20376007

[9] Gregori-Puigjané E, Setola V, Hert J, Crews BA, Irwin JJ, Lounkine E, Marnett L, Roth BL, Shoichet BK. 2012. Identifying mechanism-of-action targets for drugs and probes. Proc Natl Acad Sci USA 109:11178-83. doi:10.1073/pnas.1204524109. 22711801 PMC3396511

[10] Drews J. 2000. Drug discovery: a historical perspective. Science 287:1960–1964. doi:10.1126/science.287.5460.1960. 10720314

[11] Hage DS, Anguizola JA, Bi C, Li R, Matsuda R, Papastavros E, Pfaunmiller E, Vargas J, Zheng X. 2012. Pharmaceutical and biomedical applications of affinity chromatography: recent trends and developments. J Pharm Biomed Anal 69:93–105. doi:10.1016/j.jpba.2012.01.004. 22305083 PMC3346860

[12] Franken H, Mathieson T, Childs D, Sweetman GM, Werner T, Tögel I, Doce C, Gade S, Bantscheff M, Drewes G, Reinhard FB, Huber W, Savitski MM. 2015. Thermal proteome profiling for unbiased identification of direct and indirect drug targets using multiplexed quantitative mass spectrometry. Nat Protoc 10:1567–1593. doi:10.1038/nprot.2015.101. 26379230

[13] Martin JK, Sheehan JP, Bratton BP, Moore GM, Mateus A, Li SH-J, Kim H, Rabinowitz JD, Typas A, Savitski MM, Wilson MZ, Gitai Z. 2020. A dual-mechanism antibiotic kills Gram-negative bacteria and avoids drug resistance. Cell 181:1518–1532.e14. doi:10.1016/j.cell.2020.05.005. 32497502 PMC7780349

[14] Li X, Zolli-Juran M, Cechetto JD, Daigle DM, Wright GD, Brown ED. 2004. Multicopy suppressors for novel antibacterial compounds reveal targets and drug efflux susceptibility. Chem Biol 11:1423–1430. doi:10.1016/j.chembiol.2004.08.014. 15489169

[15] Liu A, Tran L, Becket E, Lee K, Chinn L, Park E, Tran K, Miller JH. 2010. Antibiotic sensitivity profiles determined with an Escherichia coli gene knockout collection: generating an antibiotic bar code. Antimicrob Agents Chemother 54:1393–1403. doi:10.1128/AAC.00906-09. 20065048 PMC2849384

[16] Zhu DX, Garner AL, Galburt EA, Stallings CL. 2019. CarD contributes to diverse gene expression outcomes throughout the genome of Mycobacterium tuberculosis. Proc Natl Acad Sci USA 116:13573–13581. doi:10.1073/pnas.1900176116. 31217290 PMC6613185

[17] Nonejuie P, Burkart M, Pogliano K, Pogliano J. 2013. Bacterial cytological profiling rapidly identifies the cellular pathways targeted by antibacterial molecules. Proc Natl Acad Sci USA 110:16169-74. doi:10.1073/pnas.1311066110. 24046367 PMC3791758

[18] Lamsa A, Lopez-Garrido J, Quach D, Riley EP, Pogliano J, Pogliano K. 2016. Rapid inhibition profiling in Bacillus subtilis to identify the mechanism of action of new antimicrobials. ACS Chem Biol 11:2222–2231. doi:10.1021/acschembio.5b01050. 27193499 PMC5459310

[19] Cunha BRD, Fonseca LP, Calado CRC. 2020. Metabolic fingerprinting with Fourier-transform infrared (FTIR) spectroscopy: towards a high-throughput screening assay for antibiotic discovery and mechanism-of-action elucidation. Metabolites 10:145. doi:10.3390/metabo10040145. PMC7240953 32283661

[20] Jana B, Baker KR, Guardabassi L. 2017. Macromolecule biosynthesis assay and fluorescence spectroscopy methods to explore antimicrobial peptide mode(s) of action. Methods Mol Biol 1548:181–190. doi:10.1007/978-1-4939-6737-7_12. 28013504

[21] Parsons JoshuaB, Yao Jiangwei, Frank MatthewW, Jackson Pamela, Rock CharlesO. 2012. Membrane disruption by antimicrobial fatty acids releases low-molecular-weight proteins from Staphylococcus aureus. J Bacteriol 194:5294–5304. doi:10.1128/JB.00743-12. 22843840 PMC3457211

[22] Ling LL, Schneider T, Peoples AJ, Spoering AL, Engels I, Conlon BP, Mueller A, Schäberle TF, Hughes DE, Epstein S, Jones M, Lazarides L, Steadman VA, Cohen DR, Felix CR, Fetterman KA, Millett WP, Nitti AG, Zullo AM, Chen C, Lewis K. 2015. A new antibiotic kills pathogens without detectable resistance. Nature 517:455–459. doi:10.1038/nature14098. 25561178 PMC7414797

[23] Sheldrick GM, Jones PG, Kennard O, Williams DH, Smith GA. 1978. Structure of vancomycin and its complex with acetyl-D-alanyl-D-alanine. Nature 271:223–225. doi:10.1038/271223a0. 622161

[24] Blumberg PM, Strominger JL. 1972. Isolation by covalent affinity chromatography of the penicillin-binding components from membranes of Bacillus subtilis. Proc Natl Acad Sci USA 69:3751–3755. doi:10.1073/pnas.69.12.3751. 4630162 PMC389864

[25] Sinha Roy R, Yang P, Kodali S, Xiong Y, Kim RM, Griffin PR, Onishi HR, Kohler J, Silver LL, Chapman K. 2001. Direct interaction of a vancomycin derivative with bacterial enzymes involved in cell wall biosynthesis. Chem Biol 8:1095–1106. doi:10.1016/S1074-5521(01)00075-8. 11731300

[26] Datta S, Christena LR, Rajaram YR. 2013. Enzyme immobilization: an overview on techniques and support materials. 3 Biotech 3:1–9. doi:10.1007/s13205-012-0071-7. PMC3563746 28324347

[27] Telenti A, Imboden P, Marchesi F, Lowrie D, Cole S, Colston MJ, Matter L, Schopfer K, Bodmer T. 1993. Detection of rifampicin-resistance mutations in Mycobacterium tuberculosis. Lancet 341:647–650. doi:10.1016/0140-6736(93)90417-F. 8095569

[28] Andries K, Verhasselt P, Guillemont J, Göhlmann HW, Neefs JM, Winkler H, Van Gestel J, Timmerman P, Zhu M, Lee E, Williams P, de Chaffoy D, Huitric E, Hoffner S, Cambau E, Truffot-Pernot C, Lounis N, Jarlier V. 2005. A diarylquinoline drug active on the ATP synthase of Mycobacterium tuberculosis. Science 307:223–227. doi:10.1126/science.1106753. 15591164

[29] Marcusson LL, Frimodt-Møller N, Hughes D. 2009. Interplay in the selection of fluoroquinolone resistance and bacterial fitness. PLoS Pathog 5:e10000541. doi:10.1371/journal.ppat.1000541. PMC2714960 19662169

[30] Grzesiuk E, Janion C. 1993. Some aspects of EMS-induced mutagenesis in Escherichia coli. Mutat Res Genet Toxicol 297:313–321. doi:10.1016/0165-1110(93)90022-F. 7692276

[31] Pollard JE, Snarr J, Chaudhary V, Jennings JD, Shaw H, Christiansen B, Wright J, Jia W, Bishop RE, Savage PB. 2012. In vitro evaluation of the potential for resistance development to ceragenin CSA-13. J Antimicrob Chemother 67:2665–2672. doi:10.1093/jac/dks276. 22899801 PMC3468081

[32] Wistrand-Yuen E, Knopp M, Hjort K, Koskiniemi S, Berg OG, Andersson DI. 2018. Evolution of high-level resistance during low-level antibiotic exposure. Nat Commun 9:1599. doi:10.1038/s41467-018-04059-1. 29686259 PMC5913237

[33] Baba T, Ara T, Hasegawa M, Takai Y, Okumura Y, Baba M, Datsenko KA, Tomita M, Wanner BL, Mori H. 2006. Construction of Escherichia coli K-12 in-frame, single-gene knockout mutants: the Keio collection. Mol Syst Biol 2:2006.0008. doi:10.1038/msb4100050. PMC1681482 16738554

[34] Fey PD, Endres JL, Yajjala VK, Widhelm TJ, Boissy RJ, Bose JL, Bayles KW. 2013. A genetic resource for rapid and comprehensive phenotype screening of nonessential Staphylococcus aureus genes. mBio 4:e00537-12. doi:10.1128/mBio.00537-12. 23404398 PMC3573662

[35] Nichols RJ, Sen S, Choo YJ, Beltrao P, Zietek M, Chaba R, Lee S, Kazmierczak KM, Lee KJ, Wong A, Shales M, Lovett S, Winkler ME, Krogan NJ, Typas A, Gross CA. 2011. Phenotypic landscape of a bacterial cell. Cell 144:143–156. doi:10.1016/j.cell.2010.11.052. 21185072 PMC3060659

[36] Van Opijnen T, Camilli A. 2012. A fine scale phenotype-genotype virulence map of a bacterial pathogen. Genome Res 22:2541–2551. doi:10.1101/gr.137430.112. 22826510 PMC3514683

[37] Geisinger E, Mortman NJ, Dai Y, Cokol M, Syal S, Farinha A, Fisher DG, Tang AY, Lazinski DW, Wood S, Anthony J, van Opijnen T, Isberg RR. 2020. Antibiotic susceptibility signatures identify potential antimicrobial targets in the Acinetobacter baumannii cell envelope. Nat Commun 11:6107. doi:10.1038/s41467-020-20098-z. 33235195 PMC7686321

[38] Kitagawa M, Ara T, Arifuzzaman M, Ioka-Nakamichi T, Inamoto E, Toyonaga H, Mori H. 2005. Complete set of ORF clones of Escherichia coli ASKA library (a complete set of E. coli K-12 ORF archive): unique resources for biological research. DNA Res 12:291–299. doi:10.1093/dnares/dsi012. 16769691

[39] Pathania R, Zlitni S, Barker C, Das R, Gerritsma DA, Lebert J, Awuah E, Melacini G, Capretta FA, Brown ED. 2009. Chemical genomics in Escherichia coli identifies an inhibitor of bacterial lipoprotein targeting. Nat Chem Biol 5:849–856. doi:10.1038/nchembio.221. 19783991

[40] Johnson EO, Hung DT. 2021. Using proteolytic hypomorphs to detect small molecule mechanism of action. Methods Mol Biol 2314:323–342. doi:10.1007/978-1-0716-1460-0_15. 34235661

[41] Johnson EO, LaVerriere E, Office E, Stanley M, Meyer E, Kawate T, Gomez JE, Audette RE, Bandyopadhyay N, Betancourt N, Delano K, Da Silva I, Davis J, Gallo C, Gardner M, Golas AJ, Guinn KM, Kennedy S, Korn R, McConnell JA, Moss CE, Murphy KC, Nietupski RM, Papavinasasundaram KG, Pinkham JT, Pino PA, Proulx MK, Ruecker N, Song N, Thompson M, Trujillo C, Wakabayashi S, Wallach JB, Watson C, Ioerger TR, Lander ES, Hubbard BK, Serrano-Wu MH, Ehrt S, Fitzgerald M, Rubin EJ, Sassetti CM, Schnappinger D, Hung DT. 2019. Large-scale chemical–genetics yields new M. tuberculosis inhibitor classes. Nature 571:72–78. doi:10.1038/s41586-019-1315-z. 31217586

[42] Evans JC, Trujillo C, Wang Z, Eoh H, Ehrt S, Schnappinger D, Boshoff HIM, Rhee KY, Barry CE, Mizrahi V. 2016. Validation of CoaBC as a bactericidal target in the coenzyme A pathway of Mycobacterium tuberculosis. ACS Infect Dis 2:958–968. doi:10.1021/acsinfecdis.6b00150. 27676316 PMC5153693

[43] Wang J, Soisson SM, Young K, Shoop W, Kodali S, Galgoci A, Painter R, Parthasarathy G, Tang YS, Cummings R, Ha S, Dorso K, Motyl M, Jayasuriya H, Ondeyka J, Herath K, Zhang C, Hernandez L, Allocco J, Basilio Á, Tormo JR, Genilloud O, Vicente F, Pelaez F, Colwell L, Lee SH, Michael B, Felcetto T, Gill C, Silver LL, Hermes JD, Bartizal K, Barrett J, Schmatz D, Becker JW, Cully D, Singh SB. 2006. Platensimycin is a selective FabF inhibitor with potent antibiotic properties. Nature 441:358–361. doi:10.1038/nature04784. 16710421

[44] Hart EM, Mitchell AM, Konovalova A, Grabowicz M, Sheng J, Han X, Rodriguez-Rivera FP, Schwaid AG, Malinverni JC, Balibar CJ, Bodea S, Si Q, Wang H, Homsher MF, Painter RE, Ogawa AK, Sutterlin H, Roemer T, Black TA, Rothman DM, Walker SS, Silhavy TJ. 2019. A small-molecule inhibitor of BamA impervious to efflux and the outer membrane permeability barrier. Proc Natl Acad Sci USA 116:21748–21757. doi:10.1073/pnas.1912345116. 31591200 PMC6815139

[45] Mateus A, Bobonis J, Kurzawa N, Stein F, Helm D, Hevler J, Typas A, Savitski MM. 2018. Thermal proteome profiling in bacteria: probing protein state in vivo. Mol Syst Biol 14:e8242. doi:10.15252/msb.20188242. 29980614 PMC6056769

[46] Herneisen AL, Sidik SM, Markus BM, Drewry DH, Zuercher WJ, Lourido S. 2020. Identifying the target of an antiparasitic compound in toxoplasma using thermal proteome profiling. ACS Chem Biol 15:1801–1807. doi:10.1021/acschembio.0c00369. 32597628 PMC7585386

[47] O’Rourke A, Beyhan S, Choi Y, Morales P, Chan AP, Espinoza JL, Dupont CL, Meyer KJ, Spoering A, Lewis K, Nierman WC, Nelson KE. 2020. Mechanism-of-action classification of antibiotics by global transcriptome profiling. Antimicrob Agents Chemother 64:e01207-19. doi:10.1128/AAC.01207-19. 31907190 PMC7038283

[48] Brazas MD, Hancock REW. 2005. Using microarray gene signatures to elucidate mechanisms of antibiotic action and resistance. Drug Discov Today 10:1245–1252. doi:10.1016/S1359-6446(05)03566-X. 16213417

[49] Espinoza JL, Dupont CL, O'Rourke A, Beyhan S, Morales P, Spoering A, Meyer KJ, Chan AP, Choi Y, Nierman WC, Lewis K, Nelson KE. 2021. Predicting antimicrobial mechanism-of-action from transcriptomes: a generalizable explainable artificial intelligence approach. PLoS Comput Biol 17:e1008857. doi:10.1371/journal.pcbi.1008857. 33780444 PMC8031737

[50] Zampieri M, Szappanos B, Buchieri MV, Trauner A, Piazza I, Picotti P, Gagneux S, Borrell S, Gicquel B, Lelievre J, Papp B, Sauer U. 2018. High-throughput metabolomic analysis predicts mode of action of uncharacterized antimicrobial compounds. Sci Transl Med 10:eaal3973. doi:10.1126/scitranslmed.aal3973. 29467300 PMC6544516

[51] Sinha Roy R, Yang P, Kodali S, Xiong Y, Kim RM, Griffin PR, Onishi HR, Kohler J, Silver LL, Chapman K. Direct interaction of a vancomycin derivative with bacterial enzymes involved in cell wall biosynthesis. Chem Biol. 2001 Nov; 8(11):1095-106. doi:10.1016/s1074-5521(01)00075-8. PMID: 11731300. 11731300

[52] Koul A, Dendouga N, Vergauwen K, Molenberghs B, Vranckx L, Willebrords R, Ristic Z, Lill H, Dorange I, Guillemont J, Bald D, Andries K. Diarylquinolines target subunit c of mycobacterial ATP synthase. Nat Chem Biol. 2007 Jun; 3(6):323-4. doi:10.1038/nchembio884. Epub 2007 May 13. PMID: 17496888. 17496888

[53] Gerits E, Blommaert E, Lippell A, O'Neill AJ, Weytjens B, De Maeyer D, Fierro AC, Marchal K, Marchand A, Chaltin P, Spincemaille P, De Brucker K, Thevissen K, Cammue BP, Swings T, Liebens V, Fauvart M, Verstraeten N, Michiels J. Elucidation of the Mode of Action of a New Antibacterial Compound Active against Staphylococcus aureus and Pseudomonas aeruginosa. PLoS One. 2016 May 11; 11(5):e0155139. doi:10.1371/journal.pone.0155139. PMID: 27167126; PMCID: PMC4864301. 27167126 PMC4864301

[54] Imai Y, Meyer KJ, Iinishi A, Favre-Godal Q, Green R, Manuse S, Caboni M, Mori M, Niles S, Ghiglieri M, Honrao C, Ma X, Guo JJ, Makriyannis A, Linares-Otoya L, Böhringer N, Wuisan ZG, Kaur H, Wu R, Mateus A, Typas A, Savitski MM, Espinoza JL, O'Rourke A, Nelson KE, Hiller S, Noinaj N, Schäberle TF, D'Onofrio A, Lewis K. A new antibiotic selectively kills Gram-negative pathogens. Nature. 2019 Dec; 576(7787):459–464. doi:10.1038/s41586-019-1791-1. Epub 2019 Nov 20. Erratum in: Nature. 2020 Apr;580(7802):E3. PMID: 31747680; PMCID: PMC7188312. 31747680 PMC7188312

[55] Zampieri M, Szappanos B, Buchieri MV, Trauner A, Piazza I, Picotti P, Gagneux S, Borrell S, Gicquel B, Lelievre J, Papp B, Sauer U. High-throughput metabolomic analysis predicts mode of action of uncharacterized antimicrobial compounds. Sci Transl Med. 2018 Feb 21; 10(429):eaal3973. doi:10.1126/scitranslmed.aal3973. PMID: 29467300; PMCID: PMC6544516. PMC6544516 29467300

